# Cobalt Protoporphyrin Accelerates TFEB Activation and Lysosome Reformation during LPS-Induced Septic Insults in the Rat Heart

**DOI:** 10.1371/journal.pone.0056526

**Published:** 2013-02-15

**Authors:** Kana Unuma, Toshihiko Aki, Takeshi Funakoshi, Ken-ichi Yoshida, Koichi Uemura

**Affiliations:** 1 Section of Forensic Medicine, Graduate School of Medical and Dental Sciences, Tokyo Medical and Dental University, Tokyo Japan; 2 Department of Forensic Medicine, Graduate School of Medicine and Faculty of Medicine, University of Tokyo, Tokyo, Japan; Medical College of Wisconsin, United States of America

## Abstract

Lipopolysaccharide (LPS)-induced myocardial dysfunction is caused, at least in part, by mitochondrial dysfunction. Mitochondrial dysfunction and the oxidative damage associated with it are scavenged through various cellular defense systems such as autophagy to prevent harmful effects. Our recent study has demonstrated that cobalt protoporphyrin IX (CoPPIX), a potent inducer of heme oxygenase-1 (HO-1), ameliorates septic liver injuries by enhancing mitochondrial autophagy in rats. In our current study, we show that CoPPIX (5 mg/kg s.c.) not only accelerates the autophagic response but also promotes lysosome reformation in the rat heart treated with LPS (15 mg/kg i.p.). Lysosomal membrane-associated protein-2 (LAMP2), which is essential to the maintenance of lysosomal functions in the heart, is depleted transiently but restored rapidly during LPS administration in the rat. Activation of transcription factor EB (TFEB), a master regulator of lysosomal biogenesis and autophagy, was also observed, indicating a hyper consumption and subsequent reformation of the lysosome to meet the increased demand for autophagosome cleaning. CoPPIX was found to promote these processes and tended to restore the LPS-induced suppression of cardiac performances whilst chloroquine (CQ; 20 mg/kg i.p.), an inhibitor of lysosomes and autophagic protein degradation, abrogates these beneficial effects. The cardioprotective effect of CoPPIX against LPS toxicity was also observed via decreased levels of cardiac releasing enzymes in the plasma. Taken together, our current data indicate that lysosome reformation mediated by TFEB may be involved in cardioprotection against LPS-induced septic insults, and serve as a novel mechanism by which CoPPIX protects the heart against oxidative stress.

## Introduction

Lipopolysaccharide (LPS), a major outer cell wall component of gram-negative bacteria, causes septic shock in humans and animals [1]. The administration of LPS to laboratory animals ultimately leads to multi-organ dysfunction that includes myocardial depression [2], [3], [4]. Cardiac dysfunction is caused by the production of proinflammatory cytokines, mitochondrial dysfunction, and subsequent contractile failure followed by a precipitous drop in cardiac output [2], [3], [5]. Dysfunctional mitochondria are scavenged to prevent harmful effects including apoptosis induction as well as the generation of reactive oxygen species (ROS). Autophagy has been shown to play pivotal roles in the elimination of dysfunctional mitochondria [6]. Following their elimination, dysfunctional mitochondria should be replaced to ensure that normal cellular functions are not affected. Mitochondrial reformation is mediated by transcription factors/co-factors such as nuclear respiratory factor 1 (NRF1), peroxisome proliferator-activated receptor g coactivator-1α (PGC-1α, and mitochondrial transcription factor A (TFAM) [7], [8]. Indeed, transient mitochondrial depletion followed by regeneration by these factors has been demonstrated in the hearts of LPS-treated animals as well as in cultured cardiomyocytes [9], [10], [11].

In addition to the elimination of mitochondria damaged upon pathologic insults, autophagy activation sometimes causes hyper consumption of lysosome to meet with the increased demand for cleaning the autophagosome. This would be resulted in the disturbance of lysosomal degradation and should be repaired by *de novo* synthesis of lysosomes. Yu et al. have shown previously that primary lysosomes are lost during starvation through the formation of secondary autolysosomes and are reformed to their original number after a prolonged starvation period [12]. Although the correct reformation of lysosomes may sometimes be necessary for the completion of cytoprotective autophagy processes, a sub optimal level of lysosome activity and function and subsequent impairment of autophagic flux has been reported in the pathogenesis of various disease models such as Parkinson's disease [13] and pancreatitis [14]. Transcription factor EB (TFEB) is a master regulator of lysosome biogenesis [15] and is also involved in autophagy induction [16]. Lysosome reformation through the actions of TFEB has been shown to block the pathological accumulation of autophagosomes in the brain tissue of a Parkinson's disease model animal [13]. The forced expression of TFEB also mitigates the cardiomyocyte death caused by the overexpression of pro-apoptotic BNIP3 by enhancing lysosome reformation and the subsequent resumption of the autophagic flux [17].

Heme oxygenase (HO), the rate-limiting enzyme in the heme-degradation pathway, confers cytoprotection against cellular injuries in various tissues and in various models of disease including sepsis [18]. The cytoprotective effects of HO are attributed not only to the breakdown of cytotoxic free heme but also to its catalytic products carbon monoxide (CO) and biliverdin, both of which act as antioxidants [18]. Metallo protoporphyrins, such as cobalt protoporphyrin IX (CoPPIX), zinc protoporphyrin IX (ZnPPIX), and tin protoporphyrin IX (SnPPIX), are powerful modulators of HO activities [19]. Whilst ZnPPIX and SnPPIX are HO antagonists, CoPPIX works as an inducer of HO-1, an inducible isoform of HO. Recently, we and other groups showed that the pharmacological modulation of HO affects cytoprotective autophagy during LPS administration in the liver: the HO-1 inducer CoPPIX enhances cytoprotective autophagy in the LPS-treated rat liver [20] whilst the HO antagonist SnPPIX suppresses this pathway in CLP/LPS-treated mouse liver/hepatocytes [21].

In our present study, we examined the effects of CoPPIX on the status of the mitochondria, autophagic response, and the lysosome in LPS-treated rat hearts. As observed previously in the LPS-treated rat liver [20], mitochondrial autophagy appeared to take place in the rat heart under LPS exposure. We also observed that LAMP2, an essential molecule for lysosomal functions in the heart [22], is transiently depleted during LPS administration. TFEB and the transcription of LAMP2 are activated in tandem with lysosomal depletion, suggesting that they represent a compensatory mechanism for lysosome depletion during LPS administration. The pharmacological induction of HO-1 by CoPPIX accelerates these phenomena and tends to improve the cardiac performance that has been compromised by LPS.

## Materials and Methods

### Animal experiments

The animal experimentation protocols used in this study were approved by the Institutional Animal Care and Use Committee of University of Tokyo. Five-week-old male Sprague-Dawley rats were injected intraperitoneally (i.p.) with 15 mg/kg LPS [from Escherichia coli (E. coli) obtained from Sigma (L-2630, St. Louis, MO)] dissolved in 0.5 mL isotonic NaCl, or vehicle (n = 4/group). To determine if the heart damage induced by LPS administration is attenuated by HO-1, an inducer of this enzyme, cobalt protoporphyrin IX [CoPPIX (Sigma), 5 mg/kg in 0.5 mL dimethyl sulfoxide], was injected subcutaneously into the rats 24 h before LPS treatments. Chloroquine (Wako, 20 mg/kg) or tin protoporphyrin IX [SnPPIX (Sigma), 50 mg/kg] was also injected i.p. in some experiments one or 24 h before LPS treatment, respectively. The animals in the control group received vehicle injections at the same time (n = 4/group).

### Transmission electron microscopy

The animals were anesthetized with sodium pentobarbital (Nembutal, Abbott Laboratories, North Chicago, IL) at 60 mg/kg i.p. three hours after LPS injection (15 mg/kg), and then perfused transcardially with saline, followed by treatment with a fixative solution containing 4% paraformaldehyde and 2% glutaraldehyde in 0.1 M phosphate buffer (PB), pH 7.4. After perfusion, the left ventricle of the heart was removed and sliced. After rinsing in 0.2 M PB and post-fixation with buffered 2% osmium tetroxide for 2 h, the slices were stained en bloc with saturated aqueous uranyl acetate solution for 15 min, dehydrated in a graded ethanol series, and embedded in Epon 812. Ultrathin (90 nm) sections were double stained with uranyl acetate and lead citrate and then examined by transmission electron microscopy (H-7100; Hitachi, Hitachinaka, Japan).

### Western Blotting

Samples were lysed in a buffer containing 10 mM Tris–HCl (pH 8.0), 320 mM sucrose, 1 mM EDTA, protease inhibitor cocktail (Complete, Roche, Mannheim, Germany), and phosphatase inhibitor cocktail (PhosSTOP, Roche). Protein concentrations of the sample extracts were determined using a Coomassie Protein Assay Kit (Thermo Fisher Scientific, Rockford, IL). Five µg of protein per lane was subjected to SDS-PAGE gels and then electrophoretically transferred to a PVDF membrane (Immobilon-P transfer membrane; Millipore, Billerica, MA). Western blot analysis was performed using the following antibodies: anti-microtubule-associated protein light chain 3 (LC3) (#4445, Cell Signaling Technology, Beverly, MA; 1∶5000 dilution), anti-p62/SQSTM1 (#5114, Cell Signaling Technology), anti-nuclear respiratory factor 1 (NRF1) (sc-33771, Santa Cruz Biotechnology, Santa Cruz, CA), anti-mitochondrial transcription factor A (TFAM) (sc-23588, Santa Cruz Biotechnology), anti-transcription factor EB (TFEB) (ab56330, Abcam, Cambridge, MA), anti-lysosome-associated membrane protein 1(LAMP1) (#3243, Cell Signaling Technology), anti-lysosome-associated membrane protein 2 (LAMP2) (ab37024, Abcam), anti-GAPDH (Millipore, Billerica, MA), and anti-actin (Sigma-Aldrich, St. Louis, MO; 1∶5000 dilution). Peroxidase-conjugated anti-rabbit, anti–mouse, or anti-goat IgG antibodies were obtained from Promega (Madison, WI). The target protein levels were determined from a standard curve constructed by plotting the band densities and were normalized to the actin or GAPDH levels using CS Analyzer software, ver. 3.0 (ATTO, Tokyo, Japan).

### Quantitative RT-PCR

Total RNA was prepared from rat heart using TRIzol reagent (Invitrogen, Carlsbad, CA) and reverse transcription was performed using SuperScript II reverse transcriptase (Invitrogen). Quantitative PCR was performed with a StepOnePlus Real-Time PCR System (Applied Biosystems, Foster City, CA) using SYBR green as fluorescence dye. The PCR conditions were as follows: 95°C for 20 s followed by 40 cycles of 95°C for 3 s and 60°C for 30 s. The primers used were: 5′-GCAAGGCGCTCGCCCTCAAT-3′ and 5′-GCCCGCGTGACTCCTCTTCC-3′ for LAMP1; 5′-AGCAGGTGGTTTCCGTGTCTCG-3′ and 5′-AGGGCTGCTCCCACCGCTAT-3′ for LAMP2; and 5′-CACCCGCGAGTACAACCTTCTTG-3′ and 5′-CCTCTCTTGCTCTGGGCCTCGT-3′ for actin.

### Immunohistochemical staining for anti-transcription factor EB (TEEB) and 4-hydroxy-2-nonenal (4-HNE)

Formalin-fixed, paraffin-embedded sections of rat hearts were subjected to immunohistochemical analysis for TFEB and 4-HNE as previously described. Briefly, the 3-µm thick tissue sections were deparaffinized and rehydrated, followed by microwave retrieval, according to standard procedures. The slides were sequentially blocked with avidin and biotin, and then incubated 4°C overnight with 1/100 diluted mouse anti-4-HNE antibody (Japan Institute for the Control of aging, Nikken Seil Co., Ltd, Shizuoka, Japan). Following repeat washes, the slides were incubated at 25°C for 30 minutes with biotinylated goat anti-mouse IgG (414241, 1/200 dilution; Nichirei Biosciences, Tokyo, Japan). Diaminobenzidine (DAB) was used as a substrate, and the slides were dehydrated and covered in DePex. As negative controls, control sections were incubated with mouse IgG instead of the primary antibodies at the same IgG concentrations. The slides were then analyzed under a light microscope (Olympus AX80).

### Hematoxylin and eosin (H&E) staining

The left ventricle hearts and left upper lobe of rat treated with or without LPS and/or CoPPIX were transversely cut at a 2 mm thickness, and immediately fixed in 4% paraformaldehyde and embedded in paraffin. Sections of 3-µm thickness were affixed to slides, deparaffinized, and stained with H&E to evaluate morphologic changes.

### Blood analysis

Blood samples (0.2 ml each, anticoagulant in syringe: K3-EDTA at 1.5 mg/ml) were taken from the caudal veins of the rats at 0, 3, 6, 12, 24, and 48 h after LPS injections (15 mg/kg) with or without CoPPIX treatment. The plasma aspartate aminotransferase (AST) and creatine kinase-MB (CK-MB) levels were then measured using the standard methods of SRL Inc. (Tokyo, Japan).

### Echocardiography

Rats were placed on a warm blanket, and echocardiographic parasternal long-axis images as well as M-mode tracings were obtained at the level of the papillary muscles (famio-v, model SSA-510V, Toshiba, Japan). Measurements from the long-axis views included LV dimensions at end diastole and end systole and fractional shortening. These echocardiographic measurements were made at baseline (preinjection) and at 3 and 24 h after the injection of LPS, CoPPIX, or CQ (n = 4).

### Statistical Analysis

Data are expressed as the means ± S.E. Two groups were compared using the Student's t-test. Multiple group comparisons were made using analysis of variance (ANOVA) in combination with the Tukey's or Dunnett post hoc test. For nonparametric statistical analysis, the Kruskal-Wallis test and Steel procedure were used to evaluate the differences between two groups. Differences were considered significant at P<0.05.

## Results

### Cytoplasmic vacuolization and mitochondrial damage during LPS-administration in the heart

**Figure 1:**
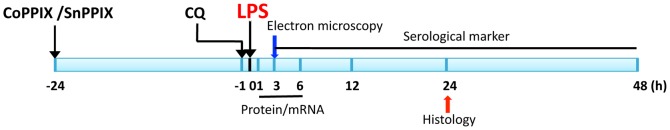
Time line illustrating the experimental design.

**Figure 2:**
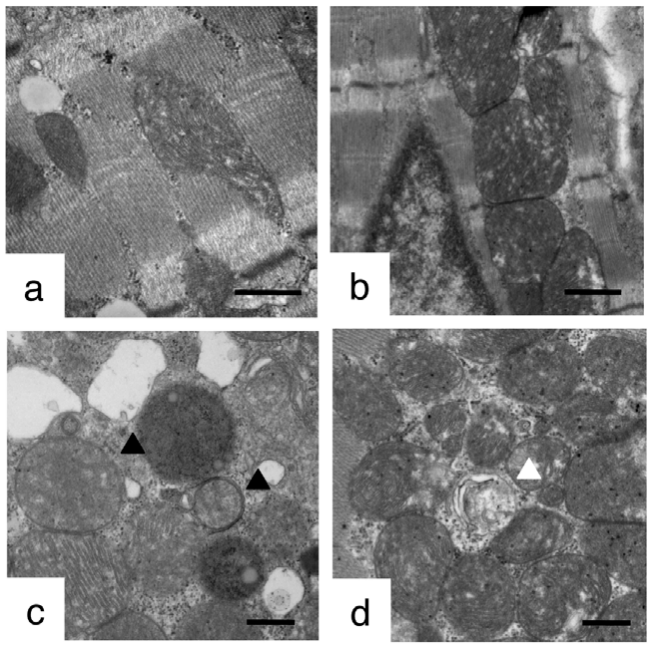
Electron microscopic analysis of the LPS-treated rat heart. Transmission electron micrographs of the rat heart treated with LPS (15 mg/kg, 3 h) with or without CoPPIX pretreatment (5 mg/kg, 24 h). Representative images in each group are shown: control (a), CoPPIX (b), LPS (c), LPS+CoPPIX (d). Bars, 500 nm. Black arrowheads indicate mitochondria swollen and/or surrounded by membranous structure. White arrowhead indicates mitochondria degraded within membranous structure.

Experimental settings of the current study are summarized in Figure 1. We first examined LPS-induced cardiomyocyte injuries. Electron microscopic analyses showed that massive cytoplasmic vacuolization was induced in the LPS-treated rat heart (Fig. 2c). In addition to the occurrence of cytoplasmic vacuoles, most of the LPS-treated rat myocardial mitochondria were swollen and severely damaged, and some mitochondria seemed to be surrounded by membranous structures (Fig. 2c). In the myocardium of LPS+CoPPIX-treated rat, some mitochondria seem to be degraded within the membranous structure (Fig. 2d), suggesting the autophagic elimination of damaged mitochondria.

### CoPPIX accelerates autophagy in response to LPS in rat heart

**Figure 3:**
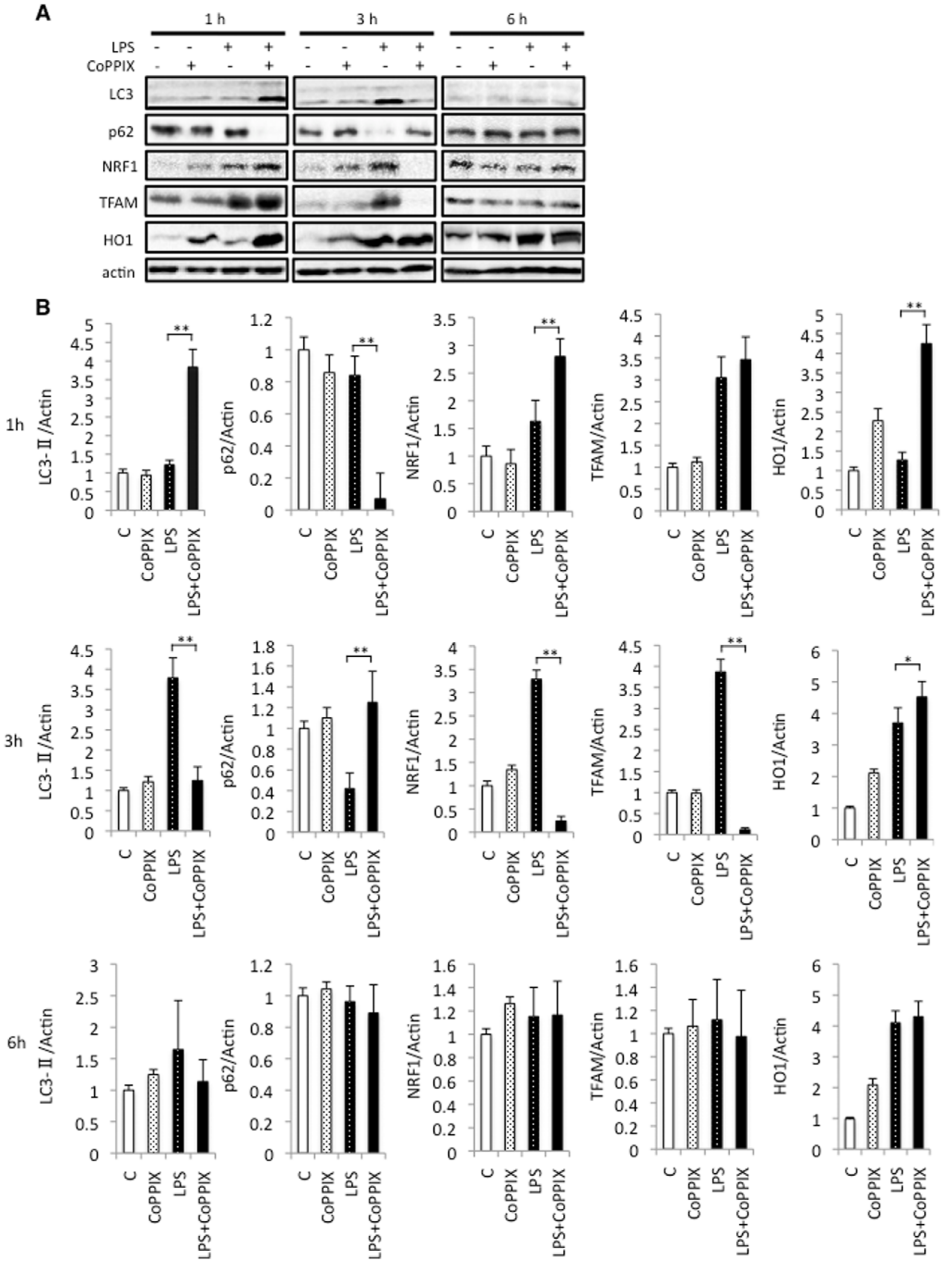
Effects of CoPPIX on autophagy and mitochondrial biogenesis during LPS treatment in the heart. A, Western blot analysis of the rat heart treated with LPS (15 mg/kg for 1, 3, and 6 h) with or without CoPPIX pretreatment (5 mg/kg, 24 h) to determine the levels of LC3-II, p62, NRF1, TFAM, and HO1. Actin was detected as a loading control. B, Ratios between LC3, p62, NRF1, TFAM, HO1, and actin determined using densitometry analysis. Each bar represents the mean ± S.E. of four animals (**p<0.01; *p<0.05). Times indicated at the leftmost in the panel show the treatment times for LPS.

As we have shown previously that pretreatment with CoPPIX accelerates LPS-induced autophagy in the rat liver [20], we also evaluated the effects of CoPPIX on LPS-induced autophagy in the heart. As shown in Figure 3, activation of LC3, a marker of autophagy, peaked at 3 hours of treatment in the LPS alone group and at 1 hour in the LPS+CoPPIX group, suggesting that CoPPIX also accelerates LPS-induced autophagy in the heart. Degradation of the autophagy substrate p62 correlated well with the activation of LC3, suggesting an increase in autophagy flux. We next examined the levels of two transcription factors, NRF1 and TFAM, since the NRF1-TFAM axis links oxidative cellular stress to mitochondrial biogenesis and has been shown to be activated during LPS treatment in the heart [7]. The NRF1-TFAM axis was found to be upregulated with faster kinetics in the LPS+CoPPIX groups than in the LPS alone group (Fig. 3A and B). Interestingly, NRF1 and TFAM are transiently depleted (3 hours after LPS) and then reformed (6 hours after LPS) in the hearts of the LPS+CoPPIX group (Fig. 3A and B). These results may indicate that the renewal of damaged mitochondria is more extensive in the LPS+CoPPIX group that in the LPS alone group. The expression of HO-1 was successfully induced in the heart by CoPPIX treatment (Fig. 3B), confirming the effect of this substance. LPS alone also induced HO-1 expression (Fig. 3B), consistent with previous reports. Increase of HO-1 in the heart of LPS+CoPPIX group was faster than that in LPS alone group, suggesting that CoPPIX should increase the cellular susceptibility to LPS (Fig. 3B).

### SnPPIX suppresses autophagy in response to LPS in rat heart

**Figure 4:**
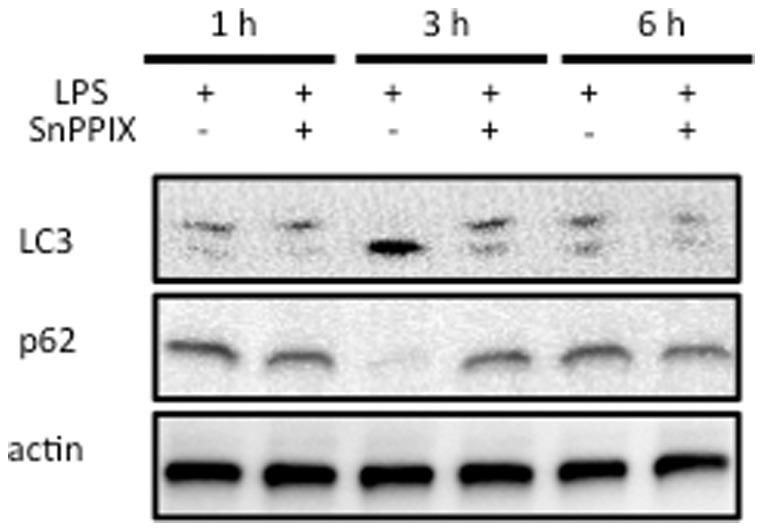
Effects of SnPPIX on autophagy during LPS treatment in the heart. Western blot analysis of the rat heart treated with LPS (15 mg/kg for 1, 3, and 6 h) with or without SnPPIX pretreatment (50 mg/kg, 24 h) to determine the levels of LC3-II and p62. Actin was detected as a loading control. Times indicated on the top of the panel show the treatment times for LPS.

To confirm the involvement of HO in the acceleration of autophagy in response to LPS, we also evaluated the effects of SnPPIX, HO inhibitor, on LPS-induced autophagy in the heart. As shown in Figure 4, neither LC3 activation nor p62 degradation was observed in the myocardium of SnPPIX+LPS-treated rat. We thus concluded that the effect of CoPPIX on autophagy is mediated through its effect on HO-1 induction.

### CoPPIX enhances transient depletion and subsequent reformation of LAMP2 in response to LPS in the rat heart

**Figure 5:**
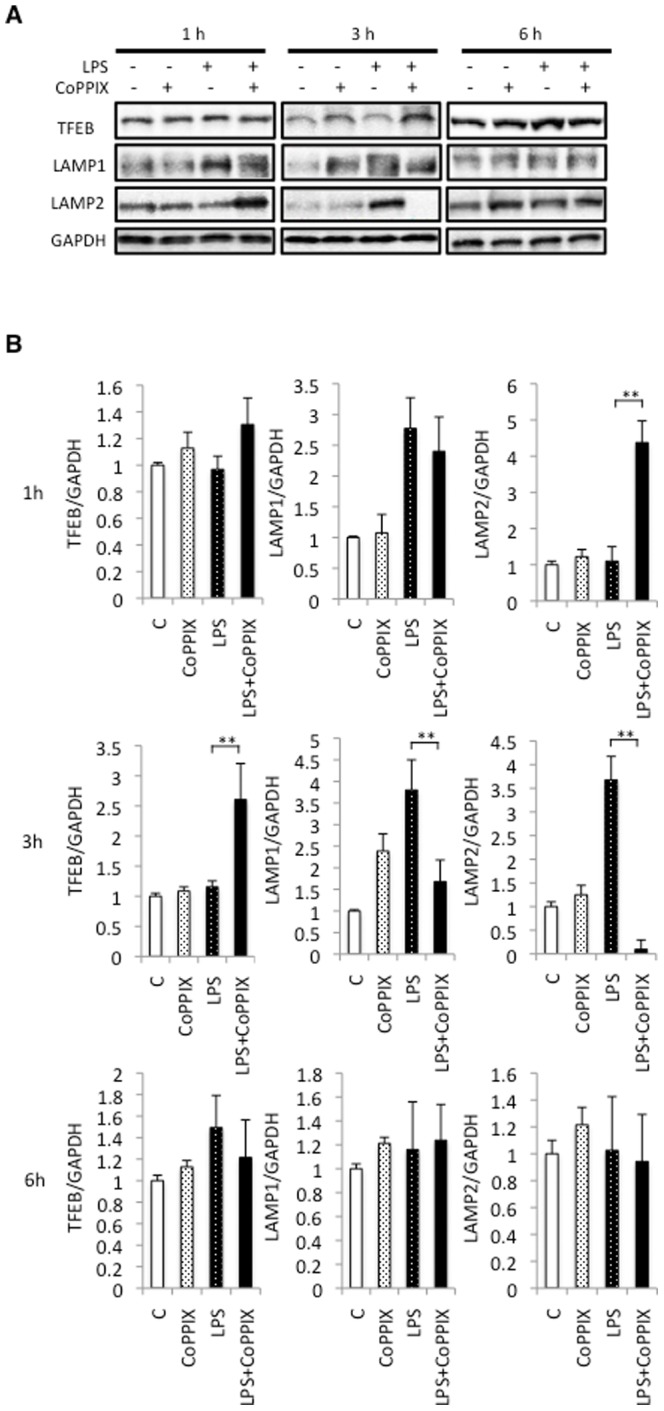
Effects of CoPPIX on lysosome reformation during LPS treatment in the rat heart. A, Western blot analysis of the rat heart treated with LPS (15 mg/kg for 1, 3, and 6 h) with or without CoPPIX pretreatment (5 mg/kg, 24 h) to determine the levels of TFEB, LAMP1, and LAMP2. GAPDH was detected as a loading control. B, Ratios between TFEB, LAMP1, LAMP2, and GAPDH determined using densitometry analysis. Each bar represents the mean ± S.E. of four animals (**p<0.01). Times indicated at the leftmost side in the panel show the treatment times for LPS.

We examined the status of the lysosomal marker proteins in our animal model. The level of LAMP1 showed the tendency to be upregulated in CoPPIX, LPS, and LPS+CoPPIX groups, but it seems to be difficult to deduce any meaning from the results. The level of LAMP2, which is involved in the elimination of autophagosome by lysosome, peaked at 3 hours of treatment in the LPS alone group and at 1 hour in the LPS+CoPPIX group (Fig. 5A and B). Like NRF1 and TFAM, LAMP2 is transiently depleted (3 hours after LPS treatment) and then reformed (6 hours after LPS treatment) in the hearts of the LPS+CoPPIX rats (Fig. 5A and B). The upregulation of TFEB was also observed in the LPS+CoPPIX hearts at 3 hours after LPS treatment (Fig. 5A and B).

### Immunohistochemical analysis of nuclear TFEB during LPS treatment

**Figure 6:**
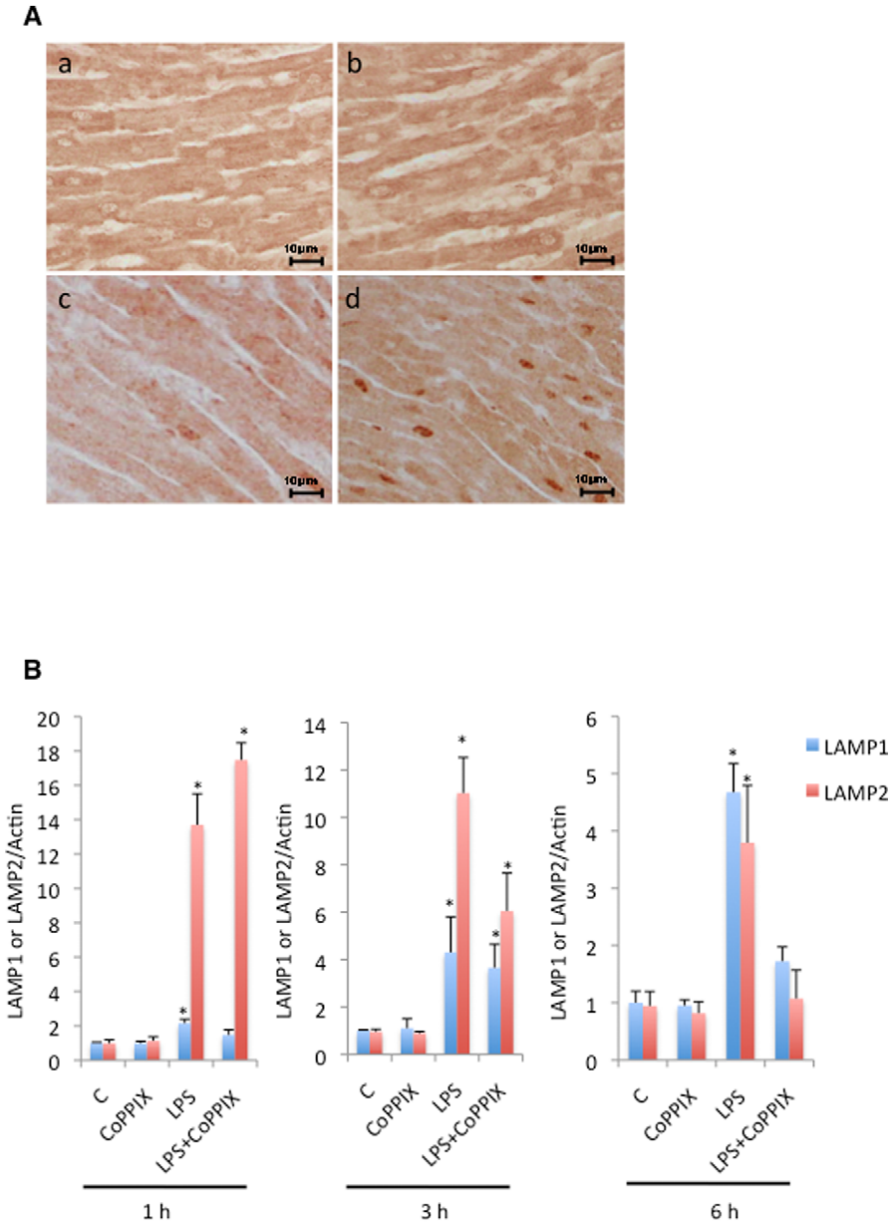
Effects of CoPPIX on nuclear translocation and activation of TFEB during LPS treatment in the rat heart. A, Immunohistochemical analysis of TFEB in the left ventricle of the rat heart at 3 h after LPS administration (15 mg/kg). Representative images in each group are shown: control (a), CoPPIX (b), LPS (c), LPS+CoPPIX (d). Bars, 5 µm. B, RT-PCR analysis of the LAMP1 and LAMP2 levels in the heart of LPS-treated and control animals for 1, 3, and 6 h. Amplification of actin cDNA was performed and served as the internal control. Each bar represents the mean ± S.E. of three animals (*p<0.05 vs. none).

We next examined the cellular localization of TFEB, as it should be translocated from the cytoplasm to the nucleus upon activation. In contrast to the slight localization of TFEB in the nucleus of the rat hearts treated with LPS alone (Fig. 6A, c), strong nuclear localization was observed in the LPS+CoPPIX treated hearts (Fig. 6A, d). The mRNA levels of LAMP1 and LAMP2, both TFEB-target genes, were also upregulated in LPS and LPS+CoPPIX groups (Fig. 6B). It seems that the levels of LAMP2 mRNA peaked within 1 hour both in the LPS alone and LPS+CoPPIX groups (Fig. 6B). These data further confirm that TFEB is activated during LPS administration. CoPPIX pretreatment accelerates this activation.

### Histological and serological evaluations of heart injuries caused by LPS-treatment in the rats

**Figure 7:**
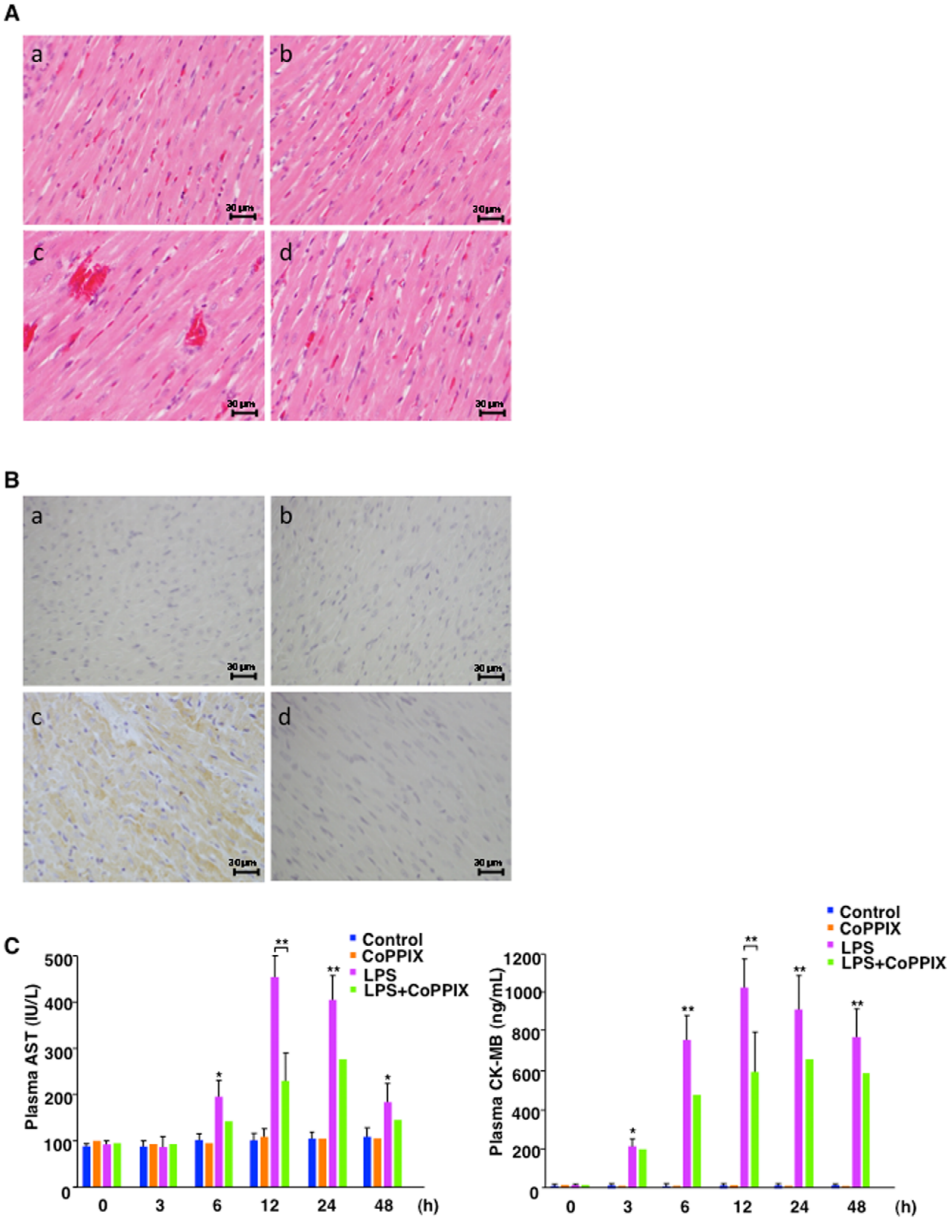
Histological and serological analyses of heart injuries during LPS administration in rats. A and B, Formalin-fixed and paraffin-embedded sections (3-µm thickness) of rat heart tissues stained with H&E and with anti-4-HNE antibody. Representative H&E stains (A, ×400) and 4-HNE stains (B, ×400) in each group (four animals) are shown: control (a), CoPPIX (b), LPS (c), LPS+CoPPIX (d) for 24 h. Bars, 30 µm. C, Plasma aspartate aminotransferase (AST, a) and creatine kinase-MB (CK-MB, b) concentrations for untreated control, CoPPIX (5 mg/kg, 24 h), LPS (15 mg/kg for indicated times), and LPS+CoPPIX groups. Values without error bars represent a single measurement while values with error bars represent mean ± S.E. of four measurements (four animals). Statistical significance was determined between LPS and LPS+CoPPIX groups (12 h) or control and LPS groups (other time points) (**p<0.01; *p<0.05).

Histological comparisons of heart sections from LPS (24 hour)-treated septic rats with matching untreated controls revealed more prominent hemorrhaging following exposure to LPS (Fig. 7A). However, cell degeneration was not evident in either treatment group (Fig. 7A). In addition, CoPPIX treatment of the rats prevented heart damage (Fig. 7A). Oxidative stress events in the heart, as assessed by examining an oxidative stress marker 4-HNE adducts, during LPS treatment showed a higher number of 4-HNE-positive cells compared with the control (Fig. 7B, c). The CoPPIX-treated group, however, showed fewer 4-HNE- positive cells after LPS treatment (Fig. 7B, d). Hence, CoPPIX protects the heart against LPS-elicited oxidative stresses. These histological observations (Fig. 7 A and B) were found in all the samples (four rats/group) (data not shown). To further confirm the heart damage, we examined the plasma concentrations of serological markers of myocardial damage. We first examined plasma levels of aspartate aminotransferase (AST), which is mostly found in the liver and heart. AST was detected in the plasma after 6 hours and was found to be increased for at least 48 hours during LPS treatment (Fig. 7C). To confirm the myocardial damage during LPS treatment, creatine kinase-MB (CK-MB), one of the most earliest and specific markers of heart damage was also examined. CK-MB was detected after 3 hours, earlier than AST, and remained to be elevated for at least 48 hours during LPS treatment (Fig. 7C). CoPPIX treatment reduced the plasma AST and CK-MB levels, confirming its protective effects against myocardial damage (Fig. 7C).

### CoPPIX ameliorates, whereas CQ exacerbates, the reduced cardiac performance caused by LPS administration

**Figure 8:**
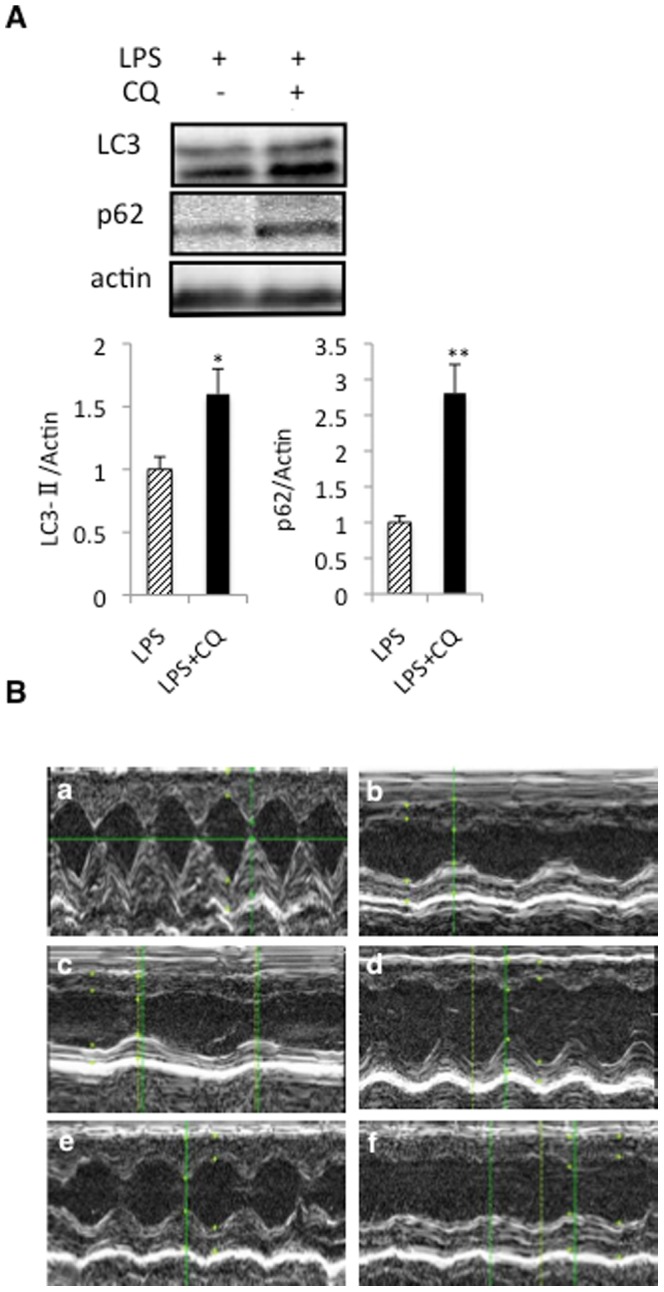
Effects of CoPPIX on left ventricular function during LPS treatment in rats. A, Western blot analysis of the rat heart with or without CQ treatment (20 mg/kg) with LPS treatment (15 mg/kg, 3 h) to determine the levels of LC3-II and p62. The ratios of LC3-II and p62 to actin were determined by densitometry analysis and are also represented. Each bar represents the mean ± S.E. of four animals. **p<0.01; *p<0.05 vs. none. B, Representative echocardiographs in each group are shown: control (a), LPS for 3 h (b), LPS+CoPPIX for 3 h (c), LPS for 24 h (d), LPS+CoPPIX for 24 h (e), and LPS+CQ for 24 h (f).

Finally, we examined effects of CoPPIX, as well as of the lysosome inhibitor CQ, on the reduced cardiac performance caused by LPS. As shown in Figure 8A, the LC3-II and p62 levels were significantly increased in the LPS+CQ group compared with the LPS alone group (Fig. 8A). Thus, CQ correctly induced lysosomal dysfunction and subsequent inhibition of autophagy flux in the rat heart. CQ also exacerbated the myocardial systolic dysfunction that was associated with LPS treatment, confirming the cardioprotective role of the autophagy/lysosome pathway in LPS-treated rats (Fig. 8B and Table 1). In contrast to the detrimental effect of CQ, pretreatment with CoPPIX tended to restore LPS-induced alterations in the myocardium (Fig. 8B and Table 1). Taken together, our current findings might indicate that CoPPIX ameliorates the left ventricular systolic dysfunction associated with LPS toxicity through enhancement of the autophagy/lysosome pathway.

**Table 1:** Echocardiographic parameters of left ventricular function were measured with LPS treatment (15 mg/kg) with or without CoPPIX (5 mg/kg) and CQ (20 mg/kg) for 3 and 24 h.

	Control	LPS (3 h)	LPS+CoPPIX (3 h)	LPS (24 h)	LPS+CoPPIX (24 h)	LPS+CQ (24 h)
HW/BW (mg/g)	3.4±0.31	3.3±0.25	3.2±0.40	3.4±0.35	3.3±0.20	3.4±0.46
LVDd (mm)	5.7±0.09	5.5±0.06	5.3±0.29	7.3±0.4**	6.8±0.3*	5.3±0.38++
LVDs (mm)	3.2±0.06	3.23±0.12	3.4±0.15	3.1±0.15	3.5±0.17	3.25±0.20
LVFS (%)	71.2±4.5	37.7±1.9**	37.7±1.5**	37.5±3.9**	48.4±3.8**	34.5±4.1+
EF (%)	96±1.7	75.1±2.2**	75.8±1.8**	76.3±3.7**	85.8±2.8*	72.3±4.5+

Shown are heart-to-body weight ratio (HW/BW), left ventricular end-diastolic diameter (LVDd), left ventricular end-systolic diameter (LVDs), left ventricular fractional shortening (LVFS), and ejection fraction (EF). Each value represents the mean ± S.E. of four animals.

** *p*<0.01;

* *p*<0.05 vs. Control.

++ *p*<0.01;

+ *p*<0.05 vs. LPS (24 h).

## Discussion

**Figure 9:**
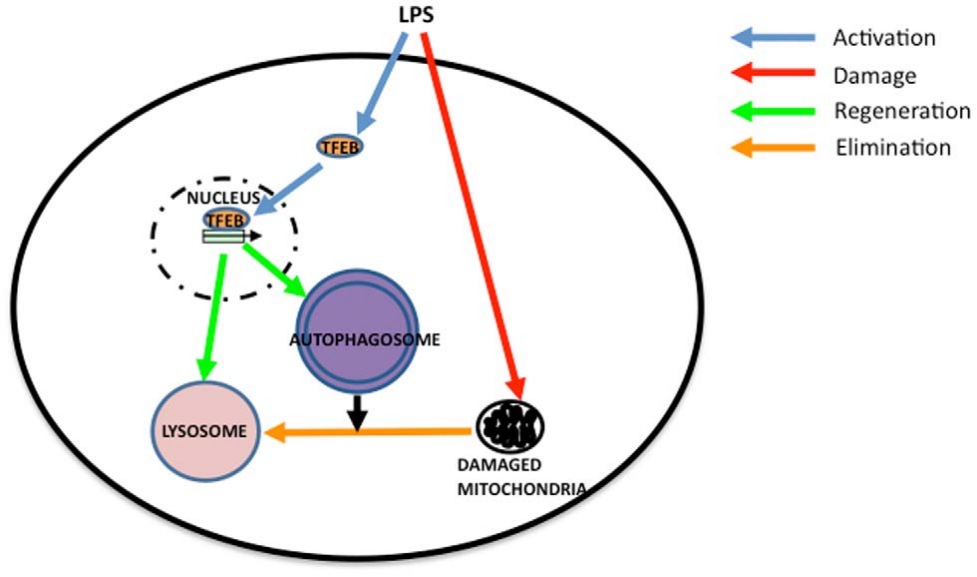
Model of organelle turnover during LPS administration in rat heart. TFEB pathway of autophagosomal and lysosomal biogenesis is activated in the heart to facilitate the elimination of damaged mitochondria during LPS administration. CoPPIX enhances the pathway and accelerates organelle turnover.

In our present study, we demonstrate the induction of autophagy in the heart during LPS administration. This process is accelerated by CoPPIX. In the heart of LPS+CoPPIX group, transient depletion and subsequent reformation of a lysosomal essential protein for autophagy, LAMP2, is observed. A master gene of lysosome biogenesis, TFEB, is also activated during LPS treatment, indicating that the TFEB activation and the subsequent reformation of lysosome are important for the protection of the heart against septic shock. Thus, our current results reveal for the first time the importance of TFEB activation in the LPS-treated heart. Results obtained in the current study are represented schematically in Figure 9.

TFEB, the somatic translocation of which is implicated in the renal carcinoma [23], is now implicated in lysosome biogenesis as well as autophagy induction [15], [16]. Mitogen-activated protein kinase (MAPK) and mammalian target of rapamycin complex1 (mTORC1) are the protein/protein complex currently known to be responsible for the regulation of TFEB [15], [16], [24], [25]. Thus, the relationship between mTORC1, MAPK, and TFEB activation during LPS insults should be examined in future studies. Interestingly, Palmieri et al., have observed that the genes involved in the recognition of pathogen associated molecular patterns, such as Toll-like receptor and Nod-like receptors, are upregulated in HeLa cells stably transfected with TFEB [26]. Therefore, the activation of TFEB might be also involved in the anti-inflammatory responses elicited by microbial pathogens, including those induced by LPS.

*LAMP1* and *LAMP2* are the genes dominantly regulated by TFEB [15], [16]. In the process of chaperone-mediate autophagy, LAMP2A, a splicing variant of LAMP2, functions as a lysosomal receptor protein that receives the cytoplasmic proteins delivered to the lysosome [27]. LAMP2 also plays a pivotal role in macroautophagy [28]. A LAMP2 deficiency in the mouse results in the accumulation of autophagic vacuoles in many organs including the heart [22], suggesting its essential role in the clearance of autophagosomes in the heart. The phenotypes of the LAMP2-deficient mouse resemble human Danon disease [29]. Thus, a LAMP2 deficiency would result in incomplete autophagy in the heart and thus a rapid activation of TFEB and subsequent recovery of LAMP2 protein during LPS treatment should be a cardioprotective reaction necessary to complete the autophagy processes. Hsieh et al., have reported in this regard that the level of LAMP1 protein is gradually decreased in the left ventricle of the CLP (cecal ligation and puncture)-induced septic mouse [30]. Subsequent lysosome shortage resulted in an incomplete cytoprotective autophagy response [30]. Rapamycin treatment rescued the CLP-induced depression of cardiac performance through the resumption of autophagic flow [30]. Although we did not observe any significant decrease in LAMP1 protein levels within the treatment times tested in our current study, the transient LAMP2 depletion in LPS+CoPPIX-treated rat heart demonstrated in our present analyses represents further evidence for the increased demand for lysosomes during septic insults in the heart. Very recently, the depletion of LAMP2 during hypoxia/reoxygenation in the mouse cardiomyocyte has been reported [31]. They also showed that adenovirus-meditated transfer of LAMP2 gene suppressed cell death during hypoxia/reoxygenation, suggesting the essential role of LAMP2 depletion and subsequent impairment of lysosomal function in the pathogenesis of ischemic heart [31].

Whereas high levels of CO inhibit mitochondrial cytochrome c oxidase activity, low levels of this compound afford cellular protection by generating low levels of reactive oxygen species (ROS) and causing a subsequent cellular adaptation that counteracts this stress event [32]. Detrimental responses due to the deficiency of HO-1 are rescued, at least in part, by the administration of CO [33]. CO has also been shown to be sufficient to induce autophagy in the mouse lung and in some cultured cells [34]. CO-releasing molecule (CO-RM) rescues mice from the CLP-induced lethal sepsis by activating mitochondrial biogenesis [35]. Although these findings suggest that CO might be sufficient to induce autophagy and mitochondrial biogenesis, we could not observe any augmentation of these processes or lysosome reformation in our CoPPIX alone treated rats (Figs. 3 and 5). Therefore, cellular reactions caused by LPS, such as ROS formation, are likely to be essential for these processes i.e. CoPPIX play roles in the augmentation/acceleration of these pathways during LPS treatment.

As shown in Figure 3A, induction of HO-1 in response to LPS was much faster in the LPS+CoPPIX group than in the LPS group. We do not know exact reason why HO-1 is induced more rapidly in the heart of LPS+CoPPIX group than LPS group. As CoPPIX has been shown to induce HO-1 expression, at least in parts, through decreasing the degradation of HO-1-inducing transcription factor Nrf2 [36]. Thus, fast induction of HO-1 during subsequent treatment with LPS in LPS+CoPPIX group might be a result of the higher availability of Nrf2. In accordance with the idea, we observed increase of Nrf2 protein in the heart of CoPPIX group (data not shown). In the current study, cardioprotective effect of CoPPIX was evaluated both histologically (Fig. 7A and B) and serologically (Fig. 7C). Besides heart, CK-MB is also found in other organs such as skeletal muscle [37]. Due to the limited tissue specificity of serological biomarkers, we also evaluated heart damage histologically. Oxidative stress by LPS and its amelioration by CoPPIX were proved in the heart (Fig. 7B). Although CoPPIX is considered to be the most potent inducer of HO among the metalloporphyrins [19], deleterious side effects have been reported for this compound [38], [39], [40]. Schmidt et al. have shown that isoflurane, an anesthetic medicine frequently used in the current clinical practice, is a potent inducer of HO-1 in the liver [40], [41]. Thus, isoflurane could potentially serve as an alternative for CoPPIX, although its effect on the heart would need to be examined. It should also be noted that injuries during LPS-induced endotoxin shock in the rat lung and liver are ameliorated by trehalose [42]. Trehalose is a potent inducer of autophagy [43] and is an activator of TFEB [15]. Hence, the beneficial effects of trehalose on septic insults might arise not only from its anti-oxidative effects but also through TFEB activation.

In conclusion, TFEB activation and subsequent lysosome reformation during septic shock in the heart is presented. Acceleration of this possible cardioprotective response by HO/CoPPIX may have novel therapeutic implications for critically ill patients with sepsis.
